# Can we encourage the endoscopic treatment for external snapping hip (ESH)? A systematic review of current concepts

**DOI:** 10.1007/s00590-024-04030-5

**Published:** 2024-06-14

**Authors:** Riccardo Giai Via, Ahmed Elzeiny, Salvatore Pantè, Simone De Vivo, Alessandro Massè, Matteo Giachino

**Affiliations:** 1 Department of Orthopaedic Surgery. Via Gianfranco, University of Turin, Centro Traumatologico Ortopedico (CTO), Zuretti 29, 10126 Turin, Italy; 2 Department of Orthopaedics and Traumatology, Faculty of Medicine, Kafr El Sheikh University, Kafr El-Sheikh, Egypt; 3 Department of Orthopaedics and Traumatology, University of Turin CTO, Via Zuretti 29, 10126 Turin, Italy

**Keywords:** External snapping hip, Hip arthroscopy, Endoscopic surgery, External snapping hip syndrome, Extra articular snapping hip, ESH

## Abstract

**Background:**

Snapping hip syndrome (SHS) is characterized by snapping sensation and pain and affects up to 10% of the general population. External snapping hip syndrome (ESHS), the most common form, is often due to repetitive movements in sports or anatomical predispositions. Conservative treatment includes physiotherapy and corticosteroid injections, while surgery is considered if conservative measures fail. Open surgical techniques carry several risks, while modern arthroscopic techniques offer less invasive options, such as endoscopic iliotibial band release (ITB) and gluteus maximus tenotomy.

**Materials and methods:**

A systematic review was conducted adhering to the PRISMA guidelines. Relevant studies were searched in four databases: Pubmed, Scopus, Embase, and Medline. The selected articles were evaluated according to the criteria of levels of evidence. The Risk of Bias In Non-randomized Studies of Interventions (ROBINS-I) was used to analyze the retrospective studies. This paper was registered in the International Prospective Registry of Systematic Reviews (PROSPERO).

**Results:**

Out of 9 included studies, 403 patients with 689 hips underwent endoscopic treatment. ITB release and his variations were the main surgical techniques. Gluteus maximus tenotomy was also used in some studies. Postoperative rehabilitation protocols varied. Patients generally experienced significant improvements in symptoms and functional outcomes, with low rates of recurrence (1.02%) and revision (0.15%). Complications were minimal.

**Conclusions:**

Endoscopic treatment of ESH shows favorable results, improving functional outcomes and returning patients to pre-injury activity levels. Long-term efficacy and costeffectiveness need to be evaluated, emphasizing the importance of large-scale prospective randomized trials to clarify surgery's benefits in refractory ESH cases.

## Introduction

Snapping hip syndrome (SHS), also known as coxa saltans, is a condition characterized by a snapping sensation, sometimes audible and/or associated with pain [1, 2]. Perrin first described this condition in 1859, and it is estimated to affect up to 10% of the general population, with a female prevalence [3]. There are two main forms based on the causes: intra-articular and extra-articular, with the latter being more common [4]. Further categorization among extra-articular forms includes internal hip snap syndrome (ISHS) and external hip snap syndrome (ESHS). ESHS, the most prevalent form, is often linked to repetitive flexion and extension movements seen in certain sports like dancing or running or to predisposing anatomical conditions such as coxa vara, prominent greater trochanter, reduced bi-iliac width, increased distance between the greater trochanters, bone spurs, and trochanteric bursa hyperplasia [5–9]. Thickening of the posterior part of the iliotibial band (ITB) or focal thickening of the anterior border of the gluteus maximus (GM) muscle can typically cause ESHS [10]. The thickened posterior ITB passes over the greater trochanter (GT), snapping anteriorly during extension and returning to a posterior position during flexion, giving rise to the phenomenon of snapping, corroborated by Ober's test positivity [7, 8, 11]. Asymptomatic snapping should be considered the usual occurrence, occurring in the 5–10% of population [12, 13]. Painful symptoms may occur in some patients due to inflammation of the GT bursa between the gluteus medius and vastus lateralis [11]. First-line treatment for such patients typically involves conservative measures such as physiotherapeutic stretching exercises, anti-inflammatory therapy, or corticosteroid infiltrative therapy targeting the greater trochanteric bursa. Surgery is indicated if conservative treatment fails for at least 6 months [13]. Historically, this surgery has been performed with an open technique with lengthening of the ITB by Z or N-plasty or with resection of a posterior portion of the ITB, elliptical resection, or step-cut procedure [2, 7, 14]. Although these techniques have shown promising results in reducing pain and clicking, they carry all the risks of open surgery, such as hematoma formation, wound complications, slow postoperative recovery, and Trendelenburg gait [15, 16]. In recent years, advancements in arthroscopic techniques have made it possible to perform surgeries such as gluteus major tenotomy and/or ITB release for this condition.

The main techniques reported in literature are ITB band release (diamond-shape defect), GM release, ITB band release (transverse cut) and ITB + GM complex release (fan-like).

ITB diamond-shape defect was developed by Ilizaliturri [17] and it consisted in one longitudinal cut along the ITB over the GT and then two transverse cuts, starting from the center of the first one and directed anteriorly and posteriorly. The resection of the four flaps created on the ITB result in the diamond-shape defect. In 2013 two more techniques have been published: ITB transverse cut and GM release.

The first one [18] is a transverse cut of ITB from anterior to posterior at the level of the snapping assessed before surgery, while the second one [1] is focused on the release of GM insertion on femoral linea aspera. The most recently described technique produces a fan-like release of ITB and GM through a stepwise approach, until the snapping is no more reproducible [19].

This systematic review aims to assess clinical outcomes, time to return to sport, and complications associated with arthroscopic surgery for external snap hip syndrome (ESHS).

## Materials and methods

### Research question

This study adhered to the Preferred Reporting Items for Systematic Reviews and Meta-Analyses (PRISMA) guidelines [20]. Two authors (RGV and AE) independently searched and evaluated the articles to avoid bias. In cases of uncertainty, a senior author (MG) was consulted to resolve doubts.

### Inclusion and exclusion criteria

Inclusion criteria for the reviewed studies were articles about patients undergoing endoscopic ITB release and/or gluteus maximus tenotomy for external hip snapping, written in English, on human subjects, published between January 2000 and February 2024 with a minimum mean follow-up of 18 months, RCTs, prospective and retrospective studies with LoE 1 to 4 [21]. Exclusion criteria were biochemical and in vitro studies, case reports, editorials, book chapters, technical reports, pre-clinical studies, and review articles. To ensure a higher standard of research quality, studies with LoE 5 were also excluded.

### Search strategy and study selection

Literature search in four databases was performed (Pubmed, Scopus, Embase and Medline) with the following MeSH terms: ((tensor fascia lata) OR (fascia lata) OR (iliotibial band) OR (tibial band)) AND ((impingement) OR (snapping)) AND ((tenotomy) OR (release)) AND ((endoscop*) OR (arthroscop*)).

The search included studies published from January 2000 to February 2024. On the 14th of February, 96 studies were found, and we declared our search completed. After the exclusion of duplicates, 43 studies were included. 32 studies were excluded, resulting in 11 eligible studies. After a full-text evaluation for eligibility, and according to the exclusion and inclusion criteria, 9 studies were selected for qualitative and quantitative analysis.

**Fig. 1:**
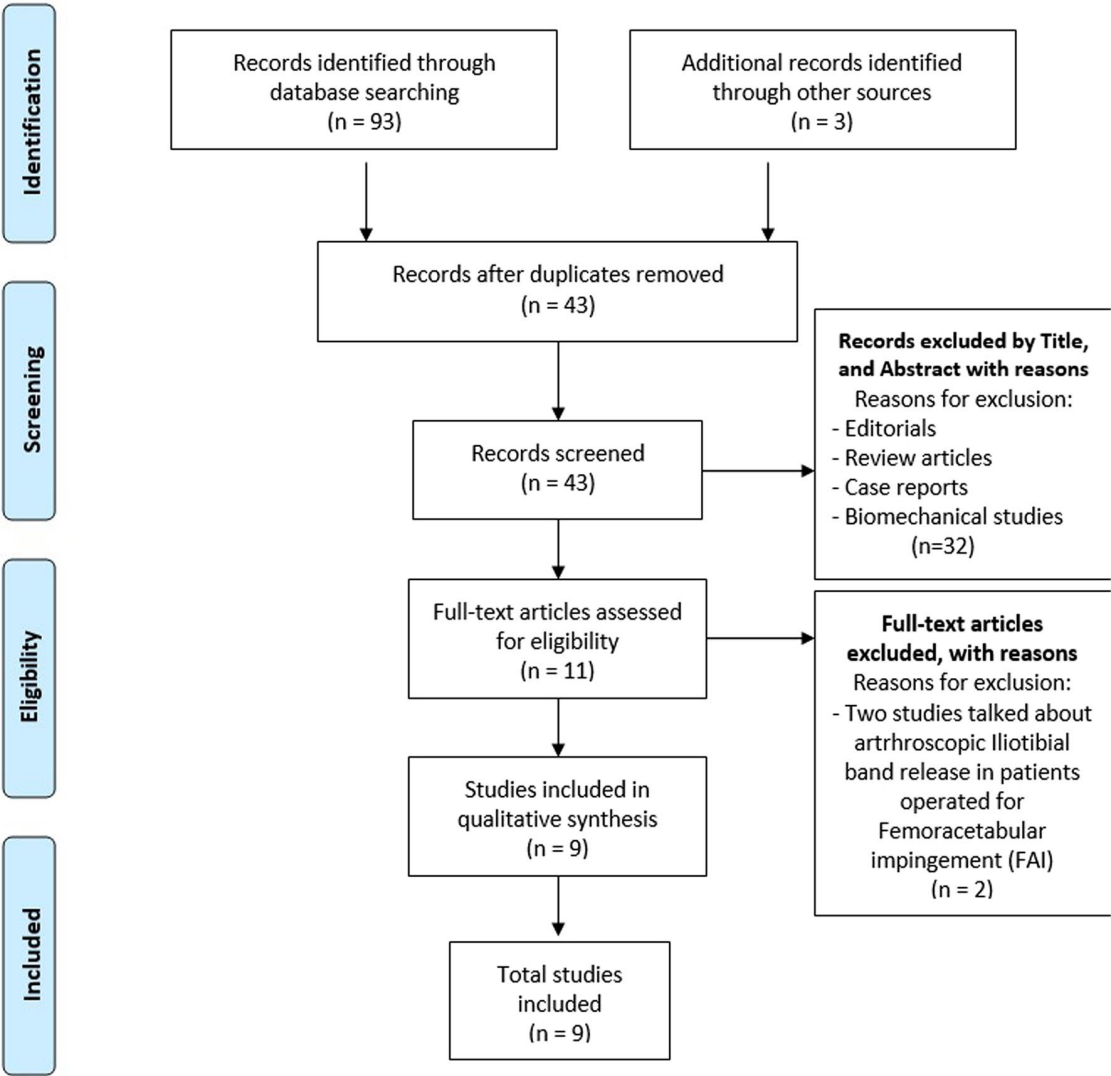
PRISMA flow diagram

The included studies reported functional outcomes, return to sport time, and complication rates among patients undergoing endoscopic release of the iliotibial band and/or gluteus maximus tenotomy. Figure 1 displays the PRISMA diagram.

### Methodological quality assessment

**Fig. 2:**
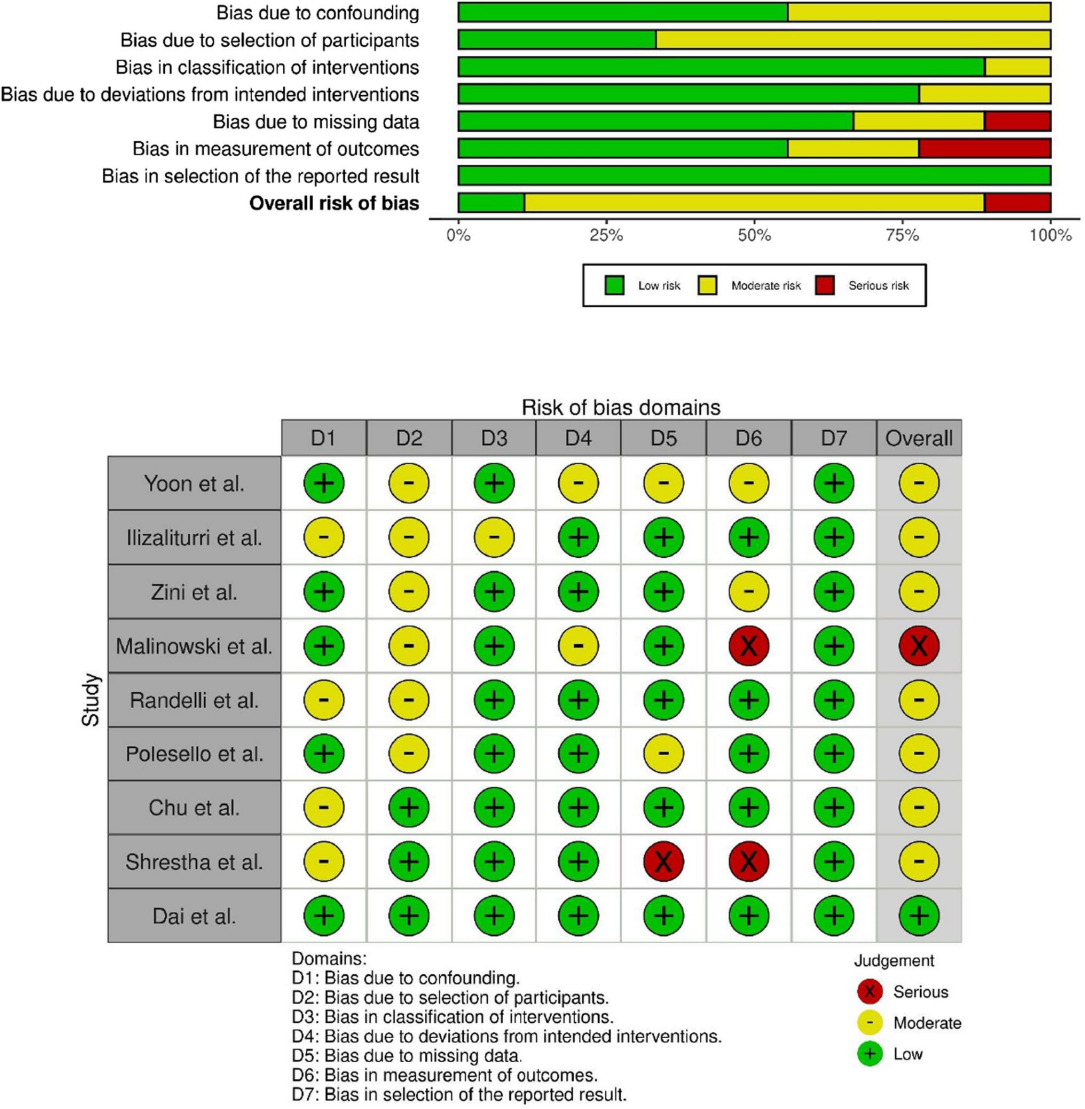
Risk of Bias In Non-randomized Studies—of Interventions (ROBINS-I) tool assessment. Risk of bias conformed by the Cochrane Handbook for Systematic Reviews of Interventions. The quality and risk of bias of individual retrospective studies included in the systematic review

Each included article underwent classification based on the Oxford Centre for Evidence-Based Medicine 2011 Levels of Evidence (LoE). With this tool, articles were classified from 1 to 5, where LoE 1 represented a better design, methodological quality, and lower risk of bias in the study under review. The included studies were analyzed with the Risk of Bias In Non-randomized Studies of Interventions (ROBINS-I) [22] (Fig. 2). Two authors (RGV, AE) used this tool, with a third author (MG) consulted to resolve uncertainties. This systematic review was registered on the International Prospective Register of Systematic Reviews (PROSPERO), CRD42024514123, in February 2024 [23].

### Data extraction

The data extracted from the included articles were systematically recorded on Excel spreadsheets by two authors autonomously and subsequently unified (RGV and AE).

The following elements were extracted from the studies: author and publication year, study design, patient sample size, mean age of the participants, mean BMI, mean follow-up time, type of anesthesia, patient positioning, time duration of surgery, rates of complications and revision, surgical details regarding the type of release of iliotibial used, scores like mHSS (modified Harris Hip Score) VAS (Visual Analogue Scale) or WOMAC (Western Ontario and McMaster Universities Arthritis Index) and Range of Motion (ROM). This enabled systematic data extraction and analysis, leading to a comprehensive understanding of study findings.

### Statistical analysis

Statistical analysis was performed using R software, (version 4.1.3, R Core Team, Vienna, Austria). Descriptive statistical analysis was applied to all data extracted from the included studies. Values of variability were extracted as standard deviation (SD) or as the range between the minimum and maximum value. For categorical variables, the absolute number and frequency of distribution were determined.

## Results

The electronic database search yielded 96 articles, of which 43 were selected for systematic review. Among them, 9 articles focused on endoscopic external snap hip (ESH) treatment, including 403 patients and 689 hips, of which 286 were bilateral, and 262 were female patients. All patients underwent ITB release, gluteus maximus tenotomy or a combination of both treatments. The information regarding the studies and patients included can be found in Table 1.

**Table 1:** Demographic data of included studies and patients

Authors	YoP	Study design (LoE)	N Patients	N Hips	Bil	Age years (range)	Male	Female	FU (months)	Symptoms (months)
Yoon et al. [18]	2014	Retrospective (III)	7	10	3	35 (25–29)	2	5	19 (12–33)	36 (24–120)
Ilizaliturri Jr. et al. [17]	2006	Prospective consecutive (IV)	10	11	1	26 (21–35)	1	9	25 (12 to 36)	31 (10–38)
Zini et al. [19]	2013	Retrospective (III)	15	15	0	25 (16–37)	3	12	33.8 (12 to 84)	18 (10–48)
Malinowski et al. [24]	2024	Retrospective cohort (III)	27	31	4	18–32	4	26	(24–56)	–
Randelli et al. [3]	2021	Retrospective (III)	22	22	0	27.9 ± 13.4 (16–76)	6	16	18 ± 9.3	–
Polesello et al. [1]	2013	Retrospective case series (IV)	8	9	1	35 (18–55)	1	7	32 (22 to 45)	36 (16–84)
Chu et al. [25]	2021	Prospective (III)	18	18	0	38.8 (21 to 52)	10	8	84 (24–204)	36 (3–120)
Shrestha et al. [26]	2017	Retrospective (III)	248	477	229	26 (8–38)	99	149	24	120 (1–360)
Dai et al. [27]	2018	Retrospective (III)	48	96	48	20.8 (18–28)	18	30	28.3 (24–48)	162 (96–228)

All studies showed failure of conservative treatment, such as physical therapy and/or corticosteroid injections, before surgery [1, 3, 17–19, 24–27].

### Surgical technique

In six studies, ITB release was the primary surgical technique, with minor variations in the shape of the release [17–19, 24–26]. Three studies employed a diamond-shaped release, one used a fan-shaped release, and another utilized a transverse release. Additionally, two studies combined ITB release with gluteus maximus tenotomy (see Table 2).

**Table 2:** Surgical technique and postoperative program of patients following arthroscopic management of external snapping hip

Authors	Position	Anesthesia	Op time (min)	Surgical technique	Post operative program
Yoon et al. [18]	Lateral	Spinal	–	ITB release (Diamond shaped defect) ± GM tenotomy	1 month: Continuous passive motion 6 weeks: Crutches with restricted active abduction
Ilizaliturri Jr. et al. [17]	Lateral	Spinal	80 (60–100)	ITB release (Diamond shaped defect)	Full weight-bearing and free hip range of motion from day 2
Zini et al. [19]	Lateral	–	–	ITB release (Transeverse cut)	15 days: Full weight-bearing with crutches 3 weeks: Quadriceps and gluteus strengthening > 3 weeks: Water physical therapy
Malinowski et al. [24]	Lateral	Spinal\General	–	"Fan-like” release of the ITB ± GM complex	2 weeks: Weight-bearing with crutches > 2 weeks: Gentle stretching
Randelli et al. [3]	Supine	General	–	GM tenotomy	3 weeks: Full weight bearing with crutches > 3 weeks: Strengthening and stretching exercises of the gluteus complex
Polesello et al. [1]	Supine	General	62 (31–97)	GM tenotomy	1 week: Full weight-bearing with crutches 3 months: Physical therapy with gluteal muscle tendon stretching
Chu et al. [25]	Lateral	General	–	ITB Diamond-shaped excision	–
Shrestha et al. [26]	Lateral	General	15–20	ITB release + TFL and GM release	Hip and knee flexion with full range of motion and crossing legs exercises
Dai et al. [27]	Lateral	Spinal/Epidural	26.41 ± 8.84	GM tenotomy	Straight line movement, squatting, sitting with stacked legs, hip abduction 3–5 times a day (20–30 reps)

Abbreviations: ITB: iliotibial band; GM:gluteus maximus; -: not specified

Three studies reported as surgical technique the gluteus maximus tenotomy. A radiofrequency device was used to identify and transect entirely the femoral insertion of the gluteus maximus tendon [1, 3, 26].

### Postoperative rehabilitation

Postoperative rehabilitation protocols varied among the studies (see Table 2). Eight studies described postoperative protocol consisting mainly of weight-bearing on crutches immediately after surgery, followed by stretching exercises. Only one study did not mention a specific postoperative rehabilitation protocol [25].

### Final reported outcomes

Five studies reported the time needed to return to preoperative activity levels, from 6 weeks to 6 months after surgery [1, 18, 24, 26, 27]. Patient-reported subjective outcome scores were used in eight studies, including measures such as patient satisfaction, the Harris Hip Score (HHS), the modified Harris Hip Score (mHHS), the Tegner Activity Level Scale, the Western Ontario and McMaster Universities Osteoarthritis Index (WOMAC), the Visual Analog Scale (VAS), and the Multicenter Arthroscopy of the Hip Outcome Research Network (MAHORN) Hip Outcome Tool (MHOT-14). All these scores reported an improvement after surgery (see Table 3).

**Table 3:** Summary of postoperative outcomes, complications, recurrences and revisions following arthroscopic management of External Hip Snapping

Authors	YoP	Preop outcome measures; mean (range)	Postop outcome measures; mean (range)	Time to return to activity	Rate of return to activity	Recurence	Revision	Complications
Yoon et al. [18]	2014	mHHS 68.2 (range, 43–73) VAS 6.8 (range, 6–9)	mHHS 94.8 (89 –100)VAS 0.2 (0–2)	6 weeks	–	0	0	1 pain
Ilizaliturri Jr. et al. [17]	2006	WOMAC 81 (78–87)	WOMAC 94 (89–96)	–	100%	1 (8.3%)	0	1 snapping
Zini et al. [19]	2013	Tegnar Activity Level 7.6 (6–9) VAS 5.5 (5–7)	HHS 97.5 (94–100) Tegnar Activity Level 7.6 (6–9) VAS 0.53 (0–2 mm) Patient Satisfaction 9.3 (8–10)	–	100%	0	0	0
Malinowski et al. [24]	2024	MHOT-14: 46	MHOT-14: 93	8 weeks	100%	0	0	5 (16.1%) buttocks asymmetry 4 (12.9%) haematoma 1 (3.2%) pain and numbness 1 (3.2%) pelvis asymmetry
Randelli et al. [3]	2021	VAS 6.8 (6–8) mHHS 48.7 (17.6–67) NAHS 49.0 (21.5–66)	VAS 0.6 (0–4) mHHS 88.2 (67–94.6) NAHS 90.8 (66–98.75)	–	100%	0	0	0
Polesello et al. [1]	2013	mHHS 61.3 ± 7.5 ( 45–70)	mHHS 77.6 ± 11.9 (62–93)	3 months	100%	2 (25%)	1 (12.5%)	2 pain
Chu et al. [25]	2021	VAS 3.67 (0–8) mHHS 70.08 (59.4–88.4)	VAS 1.17 (0–3) mHHS 93.14 (80.45–99.65)	–	–	0	0	2 tightness
Shrestha et al. [26]	2017	Add angle Type I − 14.4 ± 5.14 Type II − 31.2 ± 5.22 Type III − 49.0 ± 3.47 Type IV − 64.5 ± 4.65	Add angle Type I − 35.7 ± 4.21 Type II − 31.7 ± 2.84 Type III − 21.6 ± 3.43Type IV − 18.3 ± 3.10	6 months	–	0	0	0
Dai et al. [27]	2018	Hip adduction 5.9 ± 4.3 HHS 81.5 ± 7.2	Hip adduction 18.7 ± 4.9 HHS 99.9 ± 0.7	3 months	–	4 (4.1%)	0	1 infection

Abbreviations: YoP: Year of Pubblication; MHOT-14: MAHORN Hip outcome Tool-14; WOMAC: Western ontario and mcmaster universities osteoarthritis Index; VAS: visual analogue scale; mHHS: Modified harris hip score; NAHS: Non-arthritic hip score; -: not specified

In one study no subjective outcome measurements were applied, however it stated that patients returned to activity after 6 months without reporting recurrences, revisions, or complications. All patients' range of motion improved significantly, with higher adduction angles observed at the 24-month postoperative follow-up [26] (see Table 3). Also, in this study, 15 patients had associated knee pain due to ITB tension, resolved after surgery. Another study mentioned that two patients (4 hips) could not adduct in 90° of hip flexion or cross one leg over the other due to the intense pain. After endoscopic ITB release plus GM tenotomy, hip adduction was restored in both patients. [18]

### Complications

Patients had a low recurrence rate of pain or snapping after endoscopic management, with only 1.02% (7 of 689 hips) having a recurrence and only one revision surgery (0.15%). In the case of revision surgery, a 33-year-old male patient experienced persistent pain and clicking. After 22 months of follow-up, the patient's symptoms were resolved through an additional tenotomy of the gluteus maximus and the release of the tensor fascia lata [1].

Other postoperative complications included pain, with 0.58% of hips experiencing postoperative pain that was less annoying than preoperative symptoms. Other complications reported in studies included buttock asymmetry (16.1%), hematoma (12.9%), and pelvic asymmetry (3.2%) [24]. One study reported a 1% incidence of postoperative infection [27] (see Table 3).

## Discussion

The most important finding of this systematic review is that endoscopic management of EHS offers a safe and effective means of improving functional outcomes and returning patients to pre-injury activity levels.

The primary goal of surgery is to eliminate the snapping by releasing the ITB and along with GT bursectomy to reduce tension and to remove the inflamed tissue. Several authors have described various surgical techniques, including simple release (transverse or cross incision), release with resection, lengthening of the ITB by Z-plasty or repair, diagonal trochanteric osteotomy, cross incision with flaps sutured to the tract, resection of the posterior half of the tract at the insertion of the gluteus maximus, and ellipsoidal resection of the tract over the trochanter [28]. Open surgical approaches have been associated with complications, including recurrent snapping, persistent hip pain, and sensory nerve injury resulting from surgical incisions [29]. A study by Hoskins et al., in which 80 patients (92 hips) with ESH were operated on via an open approach, showed a failure rate of 22% [30]. Therefore, the endoscopic release was introduced to reduce complications, recurrency, and invasivity.

The success of surgery depends on the precise execution of the technique and careful patient selection, taking into account factors such as motivation and emotional stability. Surgery is an appropriate final solution for refractory cases to conservative treatment [17].

This systematic review highlights that endoscopic treatment of ESH produces favorable outcomes in the short to medium term. Endoscopic techniques demonstrate success rates ranging from 75 to 100%, with failure rates ranging from 0 to 25% (including recurrent snapping at 4%-25% and recurrent pain without snapping at 1%25%), as reported in the included studies [1, 3, 17–19, 24–27].

The study with the largest cohort (248 patients, 477 hips) reported a 100% success rate with no cases of failure or recurrence over a 2-year follow-up period [26]. Whereas the study with the least favorable outcomes involved the smallest patient sample (8 patients, 9 hips) and reported a 75% success rate with a 25% failure rate after a follow-up period of 2.6 years [1].

This review includes three distinct techniques for treating ESH. Four studies reported a diamond-shaped or transverse cut [17–19, 25] of the ITB, three studies opted for an isolated tenotomy of the gluteus maximus [1, 3, 27], while two other studies combined both techniques [24, 26].

A noteworthy finding of this review is the high rate of return to pre-injury activity levels among patients [1, 3, 17, 19, 24] (see Table 3). The time required for patients to return to pre-injury activity levels was mentioned in five studies and ranged from 6 weeks to 6 months, depending on the severity of the snapping and the specifics of the rehabilitation program. [1, 18, 24, 26, 27]

Only seven patients experienced recurrence: all but one returned to their pre-injury activity levels without the need for revision. The exceptional patient, a 33-year-old man, experienced a persistent snap and pain that needed a revision procedure, with gluteus maximus release. After a follow-up period of 22 months, the patient remained asymptomatic and snap-free [1].

The oldest study included in this review, conducted by Ilizaliturri et al. in 2006, focused on endoscopic treatment of ESH in a cohort of 10 patients (11 hips). Using the WOMAC Score, a validated joint-specific scale, the study reported a satisfactory success rate of 92 percent. In addition, the authors noted an improvement in WOMAC scores from 81 to 94 over a mean follow-up period of 22 months (range: 12–36 months) [17].

In a recent study by Malinowski et al. in 2024, 27 patients (31 hips) with ESH were evaluated after endoscopic treatment. Significant improvements were observed in the primary outcome measure, iHOT-12, which increased from 46 to 93 after a minimum of 2 years of follow-up. In addition, the study observed a 100% patient satisfaction rate, along with a low incidence of complications and return to previous activity levels within eight weeks [24].

In the most extensive study by Shrestha et al. in 2017, a retrospective evaluation was performed on a substantial cohort of patients undergoing endoscopic transverse ITB release (n = 248 patients, 477 hips). After a 2-year follow-up period, the study reported a remarkable 100% success rate with no intraoperative or postoperative complications [26].

Endoscopic surgical management of the ESH can be performed with minimal risk of recurrence and/or revision. In our systematic review, we observed similarly low incidence rates of recurrence (1.02%) and revision (0.15%).

Zini et al. conducted a retrospective evaluation in 2013 on a series of patients undergoing endoscopic transverse ITB release (n = 15 hips). After a mean follow-up of 34 months (range: 12–84 months), their cohort of patients reported no intraoperative or postoperative complications [19]. Similarly, Yoon et al. in 2014 retrospectively evaluated patients underwent endoscopic ITB release with a diamond-shaped cut with gluteus maximus tenotomy (n = 10 hips). In their cohort, only one patient reported mild pain after a mean follow-up of 19 months (range: 12–33 months) [18].

The endoscopic technique described by Ilizaliturri et al. [17] revealed satisfactory resolution of symptoms in seven of the nine hips. However, the two hips that did not have a resolution of snapping or pain showed contracture and hypotrophy of the gluteus maximus before surgery. This suggests a potentially different biomechanical etiology for these cases of snapping hips, underscoring the importance of a thorough physical examination and attention to the presence of posterior muscle contractures. In certain situations, with the recurrency of intraoperative snapping, releasing both the iliotibial band (ITB) and gluteus maximus (GM) muscles may be necessary [24].

This systematic review presents some limitations that need to be highlighted. First, a few low-quality studies are included in the analysis. Most of them are retrospective. Second, studies are susceptible to many sources of bias in data collection and reporting, participant selection, and unblinded outcomes assessment. Third, various endoscopic surgical techniques are employed. Fourth, a wide variety of follow-ups with 12–204 months were reported in the different studies. A more homogeneous and standardized clinical follow-up could improve the data's validity. However, the data presented in this review allow hypotheses to be made for future high-quality studies.

## Conclusions

External snapping hip is a condition better understood over time, but its treatment remains controversial. This systematic review shows that endoscopic treatment of external snap hip (ESH) is a viable option when conservative measures fail, mainly to relieve mechanical impingement and improve symptoms, with a low risk of complications and a high likelihood of returning patients to pre-injury activity levels. This may suggest that endoscopy may be chosen over open techniques. However, further research is essential to evaluate the long-term efficacy and cost-effectiveness of endoscopic treatment of ESH. Large-scale prospective randomized studies with carefully selected control groups are needed to clarify the potential benefits of surgery for refractory cases of ESH.

## Data Availability

Dataset analyzed in this study is available from the corresponding author on reasonable request.
